# Adjunctive treatments for pneumococcal meningitis: a systematic review of experimental animal models

**DOI:** 10.1093/braincomms/fcae131

**Published:** 2024-04-12

**Authors:** Rutger Koning, Marian A van Roon, Matthijs C Brouwer, Diederik van de Beek

**Affiliations:** Department of Neurology, Amsterdam UMC, University of Amsterdam, Amsterdam Neuroscience, 1100DD Amsterdam, The Netherlands; Department of Neurology, Amsterdam UMC, University of Amsterdam, Amsterdam Neuroscience, 1100DD Amsterdam, The Netherlands; Department of Neurology, Amsterdam UMC, University of Amsterdam, Amsterdam Neuroscience, 1100DD Amsterdam, The Netherlands; Department of Neurology, Amsterdam UMC, University of Amsterdam, Amsterdam Neuroscience, 1100DD Amsterdam, The Netherlands

**Keywords:** pneumococcal meningitis, adjunctive treatment, animal models, systematic review

## Abstract

New treatments are needed to improve the prognosis of pneumococcal meningitis. We performed a systematic review on adjunctive treatments in animal models of pneumococcal meningitis in order to identify treatments with the most potential to progress to clinical trials. Studies testing therapy adjunctive to antibiotics in animal models of pneumococcal meningitis were included. A literature search was performed using Medline, Embase and Scopus for studies published from 1990 up to 17 February 2023. Two investigators screened studies for inclusion and independently extracted data. Treatment effect was assessed on the clinical parameters disease severity, hearing loss and cognitive impairment and the biological parameters inflammation, brain injury and bacterial load. Adjunctive treatments were evaluated by their effect on these outcomes and the quality, number and size of studies that investigated the treatments. Risk of bias was assessed with the SYRCLE risk of bias tool. A total of 58 of 2462 identified studies were included, which used 2703 experimental animals. Disease modelling was performed in rats (29 studies), rabbits (13 studies), mice (12 studies), gerbils (3 studies) or both rats and mice (1 study). Meningitis was induced by injection of *Streptococcus pneumoniae* into the subarachnoid space. Randomization of experimental groups was performed in 37 of 58 studies (64%) and 12 studies (12%) were investigator-blinded. Overall, 54 treatment regimens using 46 adjunctive drugs were evaluated: most commonly dexamethasone (16 studies), daptomycin (5 studies), complement component 5 (C5; 3 studies) antibody and Mn(III)tetrakis(4-benzoicacid)porphyrin chloride (MnTBAP; 3 studies). The most frequently evaluated outcome parameters were inflammation [32 studies (55%)] and brain injury [32 studies (55%)], followed by disease severity [30 studies (52%)], hearing loss [24 studies (41%)], bacterial load [18 studies (31%)] and cognitive impairment [9 studies (16%)]. Adjunctive therapy that improved clinical outcomes in multiple studies was dexamethasone (6 studies), C5 antibodies (3 studies) and daptomycin (3 studies). HMGB1 inhibitors, matrix metalloproteinase inhibitors, neurotrophins, antioxidants and paquinimod also improved clinical parameters but only in single or small studies. Evaluating the treatment effect of adjunctive therapy was complicated by study heterogeneity regarding the animal models used and outcomes reported. In conclusion, 24 of 54 treatment regimens (44%) tested improved clinically relevant outcomes in experimental pneumococcal meningitis but few were tested in multiple well-designed studies. The most promising new adjunctive treatments are with C5 antibodies or daptomycin, suggesting that these drugs could be tested in clinical trials.

## Teaser

Koning *et al*. performed a systematic review on adjunctive treatments in animal models of pneumococcal meningitis. Fifty-eight included studies evaluated 54 treatment regiments of which 24 improved clinically relevant outcomes, although few were tested in multiple well-designed trials. The strongest evidence exists for adjunctive therapy with C5 antibodies or daptomycin.

## Graphical Abstract

**Graphical Abstract:**
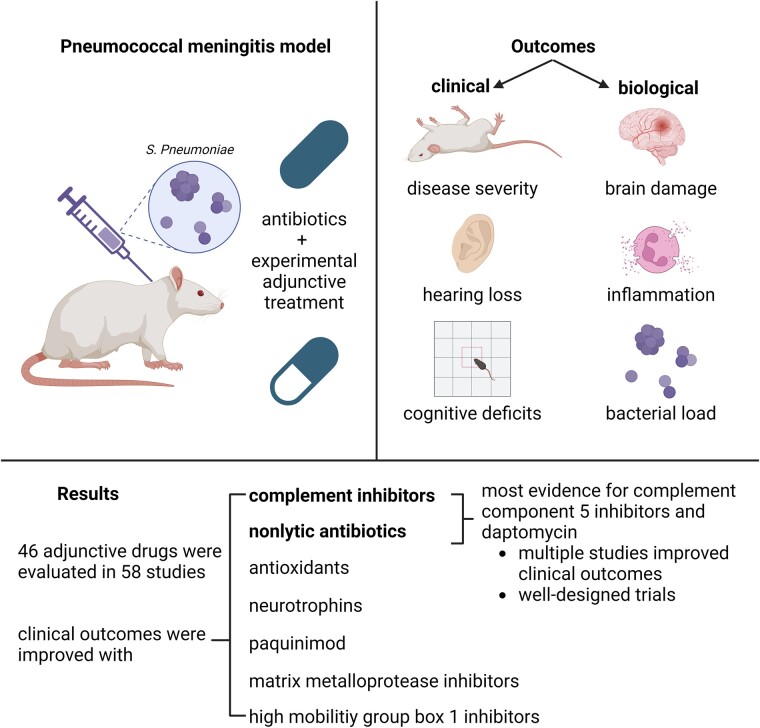

## Introduction

Bacterial meningitis is an infection of the meninges, cerebrospinal fluid (CSF) and brain, which is most commonly caused by the bacterium *Streptococcus pneumoniae* (*S. pneumoniae*).^1^ Despite substantial advances in the development of vaccines, antibiotics and anti-inflammatory treatment, pneumococcal meningitis remains a public health concern.^2,3^ Mortality rates in pneumococcal meningitis vary between 10 and 35%, and neurological sequelae, such as hearing loss and cognitive impairment, occur in up to half of survivors.^4,5^

Pneumococcal infection typically starts by nasopharyngeal colonization followed by spread to the bloodstream and eventual invasion of the subarachnoid space by passing the blood–brain barrier.^6,7^ Once in the subarachnoid space, the bacteria can multiply rapidly and trigger the activation of the immune system resulting in a massive influx of leucocytes.^6,8,9^ Although necessary to fight the infection, excessive inflammation and the release of toxic substances can also cause damage to the host, leading to brain oedema, hydrocephalus and vascular damage resulting in thrombosis or cerebral infarction.^10-12^

Urgent treatment with antibiotics results in effective bacterial killing in meningitis patients but also aggravates hyperinflammation caused by a dysregulated immune response.^13-15^ Dexamethasone, a corticosteroid with strong anti-inflammatory properties, is the only adjunctive therapy currently used in clinical practice, but has only proven efficacious in high-income countries.^16-18^ Other adjunctive treatments that have been tested in randomized controlled trials in patients include glycerol, acetaminophen or moderate hypothermia. These treatments were not proven to be beneficial or were even harmful.^19-21^

Testing new adjunctive treatments in animal studies can give an indication of the efficacy in patients and is often a pre-requisite for testing experimental therapy in humans. With this systematic review, we provide an overview of adjunctive treatments tested in animal models, to discover which new experimental treatments have the potential to improve outcomes in pneumococcal meningitis and which treatments are most likely to progress to human trials.

## Materials and methods

The literature search was performed using Medline, Embase and Scopus. We searched for studies testing adjunctive treatments in animal models of pneumococcal meningitis, published between 1 January 1990 and 17 February 2023. Search terms included: ‘animal experimentation’, ‘animal models’, ‘animals’, ‘mice’, ‘rats’, ‘rabbits’, ‘zebrafish’, ‘swine’, ‘guinea pigs’, ‘dogs’, ‘meningitis’, ‘pneumococcal meningitis’, ‘*Streptococcus pneumoniae*’ and ‘pneumococcal infection’. The full search strategy can be found in Supplementary File 1. For deduplication, the in-house developed application DedupEndnote was used.^22^

Studies were included if they fulfilled all of the following criteria: (i) meningitis was induced in animals of any species by live *S. pneumoniae*; (ii) animals received standard antibiotic treatment for meningitis, such as ceftriaxone or penicillin; (iii) the intervention group received adjunctive pharmaceutical treatment; (iv) adjunctive treatment was started concurrently with antibiotic treatment, in a time interval of 1 h prior to or after start of antibiotics; (v) antibiotic and adjunctive treatment were initiated at least 1 h after disease induction; (vi) the intervention and control groups received the same antibiotic treatment; (vii) one of the following outcome measures was reported; disease severity, hearing loss, cognitive impairment, brain injury, inflammation or bacterial load; (viii) the study was an original full paper that presented unique data; (ix) studies were published after the year 1990; and (x) studies were written in English, French, German, Spanish or Dutch.

Exclusion criteria were one of the following: (i) the use of a genetically modified animal model; (ii) the use of a genetically modified strain of *S. pneumoniae*; (iii) the use of a *S. pneumoniae* strain resistant to antibiotics used for standard clinical treatment; and (iv) meningitis is not the primarily induced disease but secondary to systemic disease. Studies evaluating dexamethasone as adjunctive treatment are included in the review but are not extensively discussed because the focus of the review is on experimental adjunctive treatments with the potential to progress to clinical trials. Dexamethasone is an established adjunctive treatment for patients with pneumococcal meningitis in high-income countries.^3^

Articles were screened by two independent researchers in two rounds using Rayyan.^23^ In the first round, studies were screened on title and abstract, in the second round on full text. Differences between researchers were resolved by discussion. Data extraction was performed by two researchers independently and compiled in an Excel file. From each study, the following data were extracted: animal species, age and sex, number of animals in experimental and control groups, inoculation method, dose and volume, pneumococcal strain, antibiotic drug and timing, adjunctive treatment drug, dose, timing and mechanism of action. The following types of outcome measures were extracted when available: (i) disease severity; (ii) hearing loss; (iii) cognitive impairment; (iv) inflammation; (v) brain injury; and (vi) bacterial load. The first three outcome parameters, disease severity, hearing loss and cognitive impairment, are clinical parameters. Disease severity includes mortality, clinical parameters such as weight loss and activity or a clinical score that integrates such parameters into one value. Hearing loss and cognitive impairment are defined as a loss of hearing or cognitive ability, respectively, measured by a functional test. The last three outcome parameters, inflammation, brain injury and bacterial load, are biological parameters. Inflammation includes measurements of inflammation such as leucocyte numbers, cytokine levels or expression of inflammatory genes. Brain injury includes damage to the brain and auditory organs as measured by biomarkers or macro- and microscopic pathology. Bacterial load is the amount of bacterial outgrowth in different body compartments. A treatment effect was defined as a significant difference in outcome between treatment and control groups as reported in the paper. Adjunctive treatments were evaluated by their effect on the defined outcomes and the quality, number and size of studies that found the effect. Clinical outcomes weighed more heavily in this evaluation than biological outcomes. To evaluate treatments despite study heterogeneity, treatments were grouped together based on mechanism of action of the adjunctive therapy. Due to study heterogeneity a statistical comparison, meta-analysis and sensitivity analysis of treatment effects were not possible. Study information was organized in Windows Excel and figures were made with Biorender.

Bias was assessed with the risk of bias tool developed by the Systemic Review Centre for Laboratory animal Experimentation (SYRCLE) by two reviewers separately.^24^ This tool evaluates selection bias, performance bias, detection bias, attrition bias and reporting bias. Studies were also evaluated for having performed a power calculation for the determination of group sizes.

We have adhered to the PRISMA 2020 reporting guidelines throughout the review (Supplementary File 2).^25^ The systematic review protocol was registered to PROSPERO (registration number: CRD42022221924).

## Results

### Study selection and description

**Figure 1:**
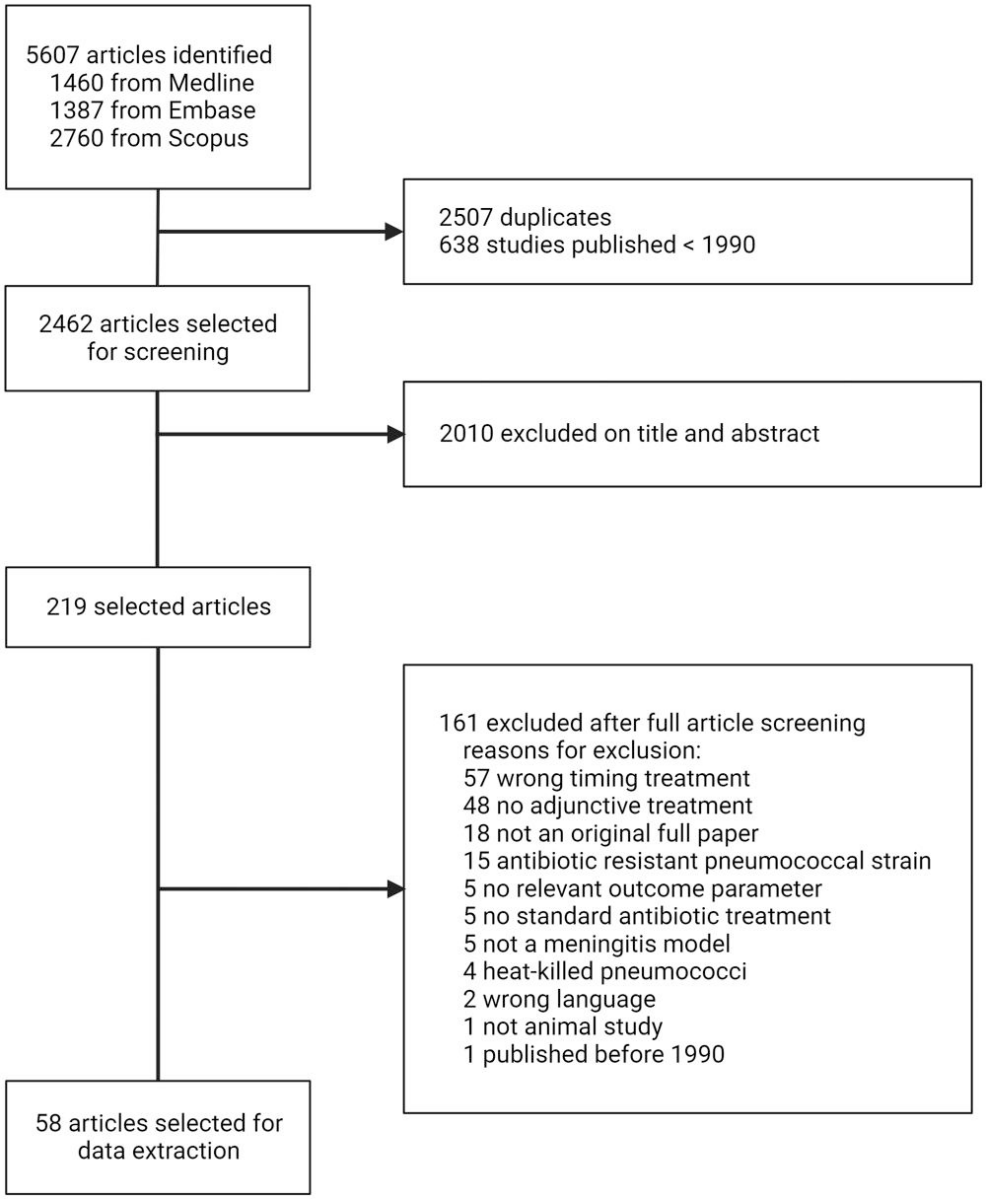
**Study selection process.** Flow chart describing the literature search and screening process of studies for this systematic review.

The literature search resulted in 2462 articles of which 219 were selected after screening on title and abstract. After full article screening, 58 studies were included for data extraction (Fig. 1, Supplementary Table 1). Twenty-nine of 58 studies (52%) used rats only, followed by 13 of 58 (22%) using rabbits, 12 of 58 (19%) used mice only, 3 of 58 (5%) used gerbils and 1 of 58 (2%) used both rats and mice (Supplementary Table 2). Age of experimental animals was described in 39 of 58 studies (67%), of which 14 studies described age non-specifically as adult. Adult animals were used in 21 of 39 studies (54%) and an infant rat model was used in 18 of 39 studies (46%, rats of 3 weeks and younger).

Animal sex was reported in 33 out of 58 studies (57%); 22 out of 33 studies (67%) used only males, 5 out of 33 studies (15%) used animals of both sexes and 6 of 33 studies (18%) used only females, all of which were performed in rabbits.

Disease was induced by direct injection of bacteria into the subarachnoid space in all studies. The most commonly used strains were of serotype 3 [39 of 58 studies (67%)] and serotype 2 [11 of 58 (19%)]. Serotypes 4 and 14 were both used in one study (2%). In 7 of 58 studies (12%), pneumococcal serotype was not described. Animals were infected with a median of 2.8 × 10^4^ of colony-forming units (CFU) [interquartile range (IQR) 1.0–16 × 10^4^, minimum 300, maximum 5 × 10^7^]. Rabbits received the highest median inoculation dose (median 3.5 × 10^5^ CFU), followed by mice (median 1.0 × 10^5^ CFU) and rats (median 2.5 × 10^4^ CFU).

**Figure 2:**
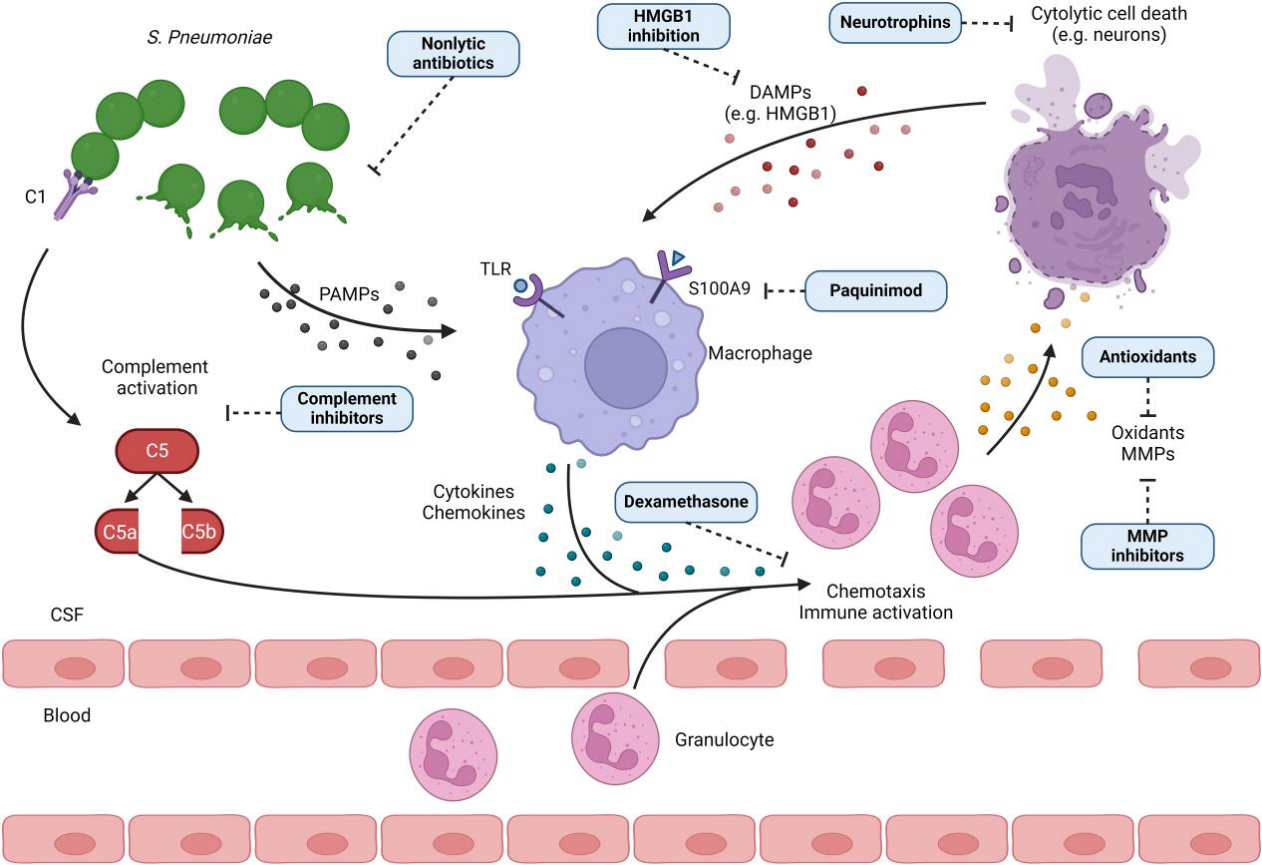
**Adjunctive therapies and their mode of action.** Mechanism of action of experimental adjunctive therapies for the treatment of pneumococcal meningitis. During meningitis, pneumococci infiltrate the subarachnoid space. The immune system is activated through recognition of pneumococcal PAMPs by pattern recognition receptors (e.g. TLRs) on resident immune cells. The release of cytokines and chemokines leads to leucocyte migration into the CSF and the production of inflammatory mediators such as reactive oxygen species and MMPs. The inflammatory reaction is amplified by the complement system, the release of additional PAMPs during pneumococcal lysis caused by the host response or antibiotic treatment, and the release of DAMPs by damaged host cells. C1, complement component 1; C5, complement component 5; CSF, cerebrospinal fluid; DAMP, danger-associated molecular pattern; HMGB1, high-mobility group box 1; MMP, matrix metalloproteinase; PAMP, pathogen-associated molecular pattern; S100A9, S100 calcium-binding protein A9; *S. pneumoniae*, *S. pneumoniae*; TLR, toll-like receptor.

Start of treatment ranged from 8 to 28 h post-infection (hpi) with a median of 18 h (IQR 16–21). Antibiotic therapy was ceftriaxone in 51 of 58 (88%) studies, 4 studies used ampicillin and 3 studies penicillin G. Adjunctive treatments were compared to therapy with antibiotics only in 50 of 58 studies (86%), and controls received antibiotic treatment in combination with dexamethasone as standard treatment in 8 out of 47 studies (17%, excluding studies where dexamethasone was the only adjunctive treatment). Studies tested a total of 46 adjuvant drugs and 54 adjuvant treatments regimens, defined as treatments with one adjuvant drug or a combination of adjuvant drugs. Dexamethasone was evaluated most often (16 studies), followed by daptomycin (5 studies) and both C5 antibody and MnTBAP were tested each in 3 studies. Nine out of 46 drugs (20%) were evaluated in 2 studies and 33 of 46 drugs (72%) were tested only once. The mechanism of action of adjunctive therapies was modulation of inflammation for 41 out of 46 drugs (87%). Neuroprotection was mentioned as the main mechanism for 5 out of 46 drugs (11%) (Fig. 2).

Inflammation as outcome was measured in 32 of 58 studies (55%), most commonly by measuring inflammatory cytokine concentrations, gene expression levels and leucocyte counts in different body compartments such as blood, CSF and brain. Brain injury was measured in 32 of 58 studies (55%), often with histopathology of the brain but also by using biomarkers of brain damage. Disease severity was evaluated in 30 of 58 studies (52%) usually by mortality or clinical scores. Hearing loss as outcome parameter was reported in 24 of 58 studies (41%) and was often tested by measuring hearing thresholds with auditory brainstem responses. Bacterial load was reported in 18 of 58 studies (31%), most often reported as CFUs in different compartments such as the CSF, brain or spleen. Cognitive impairment was tested in 9 of 58 studies (16%), with tests such as the Morris water maze or the T-maze test.

### Quality of studies

Risk of bias was assessed with the tool developed by SYRCLE (Supplementary Table 3). A lack of reporting hampered the evaluation of bias for most items. Randomization of experimental groups was performed in 39 of 58 (67%) studies, although method of randomization was mentioned in only one study. Researchers were blinded for experimental groups (=experimental design blinding) in 11 of 58 (19%) studies. Blinding during outcome evaluation (=outcome blinding) was performed in 32 of 58 (55%) studies, often for histopathology. Thirty-nine of 58 (68%) of studies gave at least limited information about baseline characteristics relevant for measured outcome parameters, such as performance on hearing tests before disease induction. Information about how animals were chosen for outcome assessment was provided in 30 of 58 (52%). It was not possible to judge bias due to allocation concealment, housing or selective outcome reporting for almost all studies due to lack of information. A power calculation for the determination of group size was only performed in 2 of 58 (3%) studies.

### Adjunctive treatment

Adjunctive treatments will be discussed together based on their mechanism of action. For the sake of brevity, only experimental adjunctive treatments evaluated in multiple studies or those with an effect on clinical outcome will be discussed here (Table 1). A complete overview of studies and outcomes can be found in Supplementary Table 4.

**Table 1:** Adjunctive treatments and their effect on outcome parameters in experimental pneumococcal meningitis

Treatment	Study	Animal	*n* ^a^	Disease severity	Hearing loss	Cognitive impairment	Inflammation	Brain injury	Bacterial load
Complement									
C5 ab	Woehrl (2)	Mouse	10/21	↓				↓	
	Kasanmoen. (1)	Mouse	32/16	↓					
C5 ab + DEX	Kasanmoen. (1)	Mouse	32/16	↓					
C5 ab + DAP^b^	Klein (2)	Mouse	20/22	↓	↓	↓	↓		=
MASP2 ab	Kasanmoen. (2)	Mouse	42/42	↓			↓		=
Human factor H	Kasanmoen. (3)	Mouse	34/33	=			↓		↑
Antibiotic									
DAP	Grandgirard	Rat	42/32	↓	↓		↓	↓	
	Muri (1)	Rat	24/42	↓				↓	
	Klein (2)	Mouse	9/13	=	↓		=		=
DAP + DEX	Klein (2)	Mouse	11/13	=	↓		=		=
DAP + cipemastat	Muri (1)	Rat	66/42	↓	↓	↓	↓	↓	↓
Doxycyclin + DAP	Muri (2)	Rat	72/72	↓	↓		↓	↓	↓
Doxycyclin	Meli	Rat	132/48	↓	↓			↓	=
Rifampicin	Spreer (2)	Rabbit	12/12	=			↓	↓	↓
	Grandgirard	Rat	38/32	=	=		↓	↓	
DAP + IL-1 ab	Klein (2)	Mouse	9/13	=	=		=		=
DAP + roscovitin	Klein (2)	Mouse	10/13	=	=		=		=
Antioxidant									
*N*-acetylcysteine	Klein (1)	Rat	5/7		↓				
	Hogen	Rat	15/11	=	↓	=			
MnTBAP	Kastenbauer (1)	Rat	6/6					↓	
	Kastenbauer (2)	Rat	11/10				↓		=
	Klein (1)	Rat	5/7		↓				
Uric acid	Kastenbauer (1)	Rat	3/6					↓	
	Kastenbauer (2)	Rat	8/10				↓		=
ASC	Kastenbauer (2)	Rat	4/10				=		=
Uric acid + ASC	Kastenbauer (2)	Rat	6/10				↓		=
SPD	Ge (2004)	Gerbil	20/10		=				
P0801	Barichello (2)	Rat	5–7				↓		
HMGB1 inhibition									
Box A protein	Hohne	Mouse	8/10	↓			↓	↓	=
Ethyl pyruvate	Hohne	Mouse	9/9	↓			↓	↓	
HMGB1 ab	Masouris	Mouse	8/12	↓			↓	↑	=
HMGB1 + DEX^b^	Masouris	Mouse	8/12	=			↓	=	=
HMGB1 + PAQ	Masouris	Mouse	6/9	=			↓	=	↑
Neurotrophin									
BDNF	Li	Rat	8/8		↓				
	Song	Rat	16/16		=			↓	
Neutrotrophin-3	Demel	Mouse	11/16	=	↓			=	
LM11A-32	Zhang	Rat	14/14	=			↓	↓	
Matrix metalloproteinase inhibition									
Cipemastat	Muri (1)	Rat	24/42	↓				↓	
B-1101	Leib (1)	Rat	48/56			↓		↓	
GM6001	Liu	Rat	24/24	↓		↓		↓	
Paquinimod									
PAQ	Wache	Mouse	9/9	↓			↓		=
	Masouris	Mouse	8/9	↓			↓	=	=

Experimental adjunctive treatments discussed in the results are shown. A downward arrow (↓) indicates that the outcome parameter was significantly lower in the treated group. Equal sign (=) indicates that there was no significant difference in outcome parameter between treatment and control groups. An upward arrow (↑) means that the outcome parameter was significantly higher in the treatment group. An empty box means that this outcome has not been measured in the study. Ab, antibody; ASC, ascorbate; BDNF, brain-derived neurotrophic factor; C5, complement component 5; DAP, daptomycin; DEX, dexamethasone; HMGB1, high-mobility group box 1; MASP2, mannose-binding protein-associated serine protease 2; MnTBAP, Mn(III)tetrakis(4-benzoicacid)porphyrin chloride; PAQ, paquinimod; SPD, superoxide dismutase.

^a^Number of animals per group (intervention/controls).

^b^Compared to ceftriaxone + dexamethasone therapy.

### Antibiotics

Standard treatment for pneumococcal meningitis involves antibiotics that kill and lyse bacteria, releasing bacterial components into the CSF that can contribute to inflammation.^2^ Adjunctive treatment with non-lytic, bacteriostatic antibiotics can prevent the release of pathogen-associated molecular patterns and limit the inflammatory reaction (Fig. 2).^14^ Six studies evaluated the use of the non-lytic antibiotics daptomycin, doxycycline and rapamycin as adjunctive treatment (Table 1).^26-31^

Daptomycin as sole adjunctive treatment was evaluated in three randomized studies, two of which were blinded.^26,27,29^ The first study in 74 infant rats (42 intervention, 32 control) showed that daptomycin reduced weight loss in the acute phase of disease, reduced hearing loss, reduced CSF inflammation and decreased neuronal apoptosis on histopathology.^26^ The second study including 66 infant rats (24 intervention, 42 control) showed a small but significant improvement in clinical scores (8%) and confirmed the protective effect of treatment on neuronal apoptosis.^27^ Finally, a randomized and blinded study with 22 mice (9 intervention, 13 control) also found a protective effect of daptomycin on hearing loss, although mortality and CSF inflammation were comparable between groups.^29^ Combining daptomycin with other adjuvant drugs further improved outcomes: daptomycin in combination with doxycycline reduced mortality from 86% to 64% in a randomized, blinded trial including 144 animals (72 intervention, 72 control), decreased levels of pro-inflammatory interleukin-1 beta (IL-1beta) and interleukin-6 (IL-6) in the CSF and reduced cortical necrosis.^28^ Furthermore, combination treatment of daptomycin with either C5 antibody or cipemastat resulted in improved disease severity, hearing or cognition (see ‘Complement system’ and ‘Matrix metalloproteinase’).^27,29^

Doxycycline as adjunctive treatment was assessed in one large, randomized study with 180 infant rats (101 intervention, 79 control). Mortality was reduced from 48% to 20% and hearing loss was reduced in combination with a decreased loss of cochlear neurons and cortical necrosis.^30^ Modest benefits were seen for adjunctive treatment with the non-lytic antibiotic rifampicin in two studies, the first using 24 rabbits (12 intervention, 12 control) and the second 70 infant rats (38 intervention, 32 control).^26,31^ The studies demonstrated a neuroprotective effect and decreased CSF inflammation, but without improving clinical parameters such as mortality and hearing.

### Oxidative stress

Reactive oxygen and nitrogen species are generated during inflammation and can contribute to tissue injury (Fig. 2).^32-38^ Reducing oxidative stress during pneumococcal meningitis through adjunctive treatment has been tested in six studies with six different drugs.^39-44^  *N*-acetylcysteine (NAC) treatment was evaluated in two studies.^39,40^ The first used 12 rats (7 intervention, 5 control) in a non-randomized non-blinded trial and found a protective effect on hearing and cochlear histopathology.^39^ The second study included 26 mice (15 intervention, 11 control) in a blinded trial and confirmed the protective effect on hearing, but found no improvement on disease severity or cognition.^40^

MnTBAP is a metalloporphyrin, a class of catalytic antioxidants that do not only scavenge reactive oxygen species but also modulate reactive-species-based redox signalling pathways.^45,46^ MnTBAP was evaluated in three small non-randomized non-blinded studies using rats.^39,41,42^ Treatment with MnTBAP decreased cochlear blood-labyrinth disruption in one study with 12 rats (6 intervention, 6 control) and decreased leucocyte infiltration and inflammatory cytokine levels in a second study with 21 rats (11 intervention, 10 control).^41,42^ The third study looked specifically at the effect on hearing in 12 rats (5 intervention, 7 controls) and found that MnTBAP protected against hearing loss.^39^

Other antioxidant drugs were tested in small non-randomized and unblinded trials, in which clinical parameters were either not investigated or not improved. Uric acid was given to rats and reduced CSF inflammation in one study and reduced blood-labyrinth permeability in the second.^41,42^ Superoxide dismutase protected gerbils against cochlear fibrosis, but failed to reduce hearing loss.^44^ The antioxidant P0801 decreased oxidative stress in rats, but was not evaluated for other outcome parameters.^43^ Finally, ascorbate was given to rats but did not improve inflammation or brain damage.^42^

### Complement system

The complement system is part of the innate immune system involved in clearing pathogens and amplifying the immune response during infection (Fig. 2).^47^ The complement cascade can be activated by the classical, alternative or lectin pathway, after which the final common pathway is activated by cleavage of C5.^48^

Three studies evaluated adjuvant treatment with antibodies against C5.^29,49,50^ The first study used 31 mice (10 experimental treatment, 21 controls) in a non-blinded non-randomized trial.^49^ Therapy resulted in a reduction of mortality from 33% to 0% and a decreased rate of cerebral damage. Next, in a randomized and blinded study, mice were treated adjunctively with C5 antibodies (*n* = 32), dexamethasone (*n* = 16), C5 antibodies and dexamethasone (*n* = 32) or saline (*n* = 16, controls).^50^ Combined treatment with dexamethasone and C5 antibodies reduced mortality from 100% to 60% and improved clinical scores compared to treatment with ceftriaxone alone. Treatment with C5 antibodies also reduced mortality when compared to all treatments without C5 antibodies from 90% to 71%. Using a linear mixed model, a slower increase of clinical scores was found for both dexamethasone and C5 antibody treatment independently. Finally, a randomized blinded study tested treatment with C5 antibodies and daptomycin in 42 mice (20 experimental treatment, 22 controls).^29^ Treatment improved clinical scores in the acute phase by 78% compared to adjuvant treatment with either dexamethasone or daptomycin. Additionally, both hearing and cognition were improved, and CSF leucocyte infiltration was decreased.

Smaller beneficial effects were seen when other components of the complement system were targeted for adjunctive treatment. Treatment with antibodies against mannose-binding lectin-associated serine protease 2 (MASP2), part of the lectin activation pathway, was evaluated in one randomized blinded study with 84 mice (42 intervention, 42 control).^51^ Experimental therapy decreased tumour necrosis factor (TNF) plasma levels and delayed the increase in clinical scores. Human factor H, a regulator of the alternative pathway, was given as adjunctive treatment in another randomized blinded study with 67 mice (34 intervention, 33 control).^52^ Although complement activation was inhibited, bacterial outgrowth was increased and therapy did not improve disease severity or mortality.

### HMGB1

HMGB1 is a damage-associated molecular pattern (DAMP) secreted during immune cell activation or cell death (Fig. 2).^53,54^ By binding to TLR4 and receptor for advanced glycation endproducts (RAGE) receptors, HMGB1 can contribute to inflammation and inhibition of HMGB1 has been experimentally investigated as treatment for sepsis and cerebral ischaemia.^55-57^ Two studies have evaluated inhibition of HMGB1 as strategy for adjunctive therapy in pneumococcal meningitis.^58,59^

Ethyl pyruvate and Box A protein, two drugs that inhibit HMGB1, were tested in a small non-randomized non-blinded study.^58^ Mice were treated with ethyl pyruvate (*n* = 8), Box A protein (*n* = 9) or the respective vehicles as control (*n* = 10 and *n* = 9). Both treatments decreased leucocytes infiltration into the CSF and reduced cerebral haemorrhages. Clinical scores at 2 days after infection improved with 38% and 54% for treatment with Box A protein and ethyl pyruvate, respectively.

HMGB1 was specifically targeted with anti-HMGB1 antibody in a randomized study using mice.^59^ Experimental groups were treated with anti-HMGB1 antibody (*n* = 8), HMGB1 antibody and dexamethasone (*n* = 9) or anti-HMGB1 isotype antibodies as controls (*n* = 12). This study also showed a positive effect of HMGB1 inhibition on clinical scores and CSF leucocyte infiltration, but led to an increase in cerebral haemorrhages. Although the mechanism behind this side effect is unknown, mice treated with isotype antibodies did not suffer from increased bleeding suggesting that it is caused by a specific interaction of anti-HMGB1 with its target. Increased bleeding was prevented by combining anti-HMGB1 with dexamethasone.

### Matrix metalloproteinase

MMPs are a family of enzymes involved in the degradation of extracellular matrix proteins, membrane receptors, cytokines and growth factors.^60^ MMPs are released during pneumococcal meningitis and contribute to inflammation (Fig. 2).^61,62^ Three studies using infant rats tested adjunctive treatment with three MMPs inhibitors: BB-1101, GM6001 and cipemastat.^27,63,64^

Treatment with BB-1101, a broad-spectrum inhibitor of MMPs and TNF, was evaluated in a randomized non-blinded study with 104 infant rats (48 intervention, 56 control).^63^ Treatment protected against cortical damage and hippocampal apoptosis. Learning, measured three weeks after infection by completion of a water maze, was also improved in treated rats.

GM6001, a broad-spectrum inhibitor of MMPs, was tested in a randomized non-blinded study with 48 infant rats (24 intervention, 24 controls) and improved clinical scores, protected against both cortical damage and hippocampal apoptosis and improved learning and memory.^64^

Cipemastat, a selective inhibitor of matrix metalloproteinase-1 (MMP-1), was tested in a randomized blinded trial with 132 infant rats. Infant rats received either cipemastat (*n* = 24), cipemastat and daptomycin (*n* = 66) or vehicle as control (*n* = 42).^27^ Cipemastat treatment reduced hippocampal apoptosis and clinical scores improved by 10% 2 days after infection. Combining cipemastat with daptomycin resulted in a quicker improvement of clinical scores and also improved hearing, learning and memory.

### Neurotrophin

Neurotrophins are growth factors that promote neuronal growth and survival through binding to cell surface receptors.^65^ Treatment with neurotrophins or modulation of downstream pathways could potentially reduce meningitis-induced neuronal apoptosis and cell death (Fig. 2).

Brain-derived neurotrophic factor (BDNF) is a well-studied neurotrophin with roles in neuronal survival and differentiation.^66,67^ Two studies tested BDNF as adjunctive treatment by delivering it through a cannula implanted into the cerebral ventricles.^68,69^ The first study started with adjunctive BDNF treatment at 24 hpi in eight infant rats and compared them to eight placebo-treated controls.^68^ Seven days after infection, hearing thresholds were significantly decreased in the treated group, indicating reduced hearing loss. The second study evaluated the effect of BDNF treatment on neuronal damage and hearing in eight treated and eight control infant rats.^69^ At 7 and 28 dpi, there was an increase in surviving neurons in the frontal cortex, although hearing measured with brainstem auditory evoked potentials was not improved.

Whereas BDNF stimulates neuronal survival, its precursor proBDNF has the opposite effect and induces neuronal apoptosis and death through binding to the tumour necrosis factor receptor molecule p75 neurotrophin receptor (p75NTR).^70-72^ Blocking this interaction with LM11A-31, a modulator of p75NTR, has anti-inflammatory and neuroprotective effects in animal models of Alzheimer’s disease and Huntington’s disease.^73,74^ One study tested treatment with LM11A-32 in an infant rat model of pneumococcal meningitis.^75^ In pre-treatment experiments clinical severity, histopathological injury and neuroinflammation were reduced. Adjunctive treatment was tested in 28 infant rats (14 intervention, 14 control) that were followed for 1–4 weeks. Treatment decreased leucocyte infiltration in the brain on histology and decreased hippocampal expression of inflammatory cytokines. Additionally, hippocampal neurogenesis was improved as shown by an increased number of neuronal precursor cell and mature neurons. However, treatment did not improve survival or clinical severity.

Another member of the neurotrophin family, neurotrophin-3, was evaluated in one study using mice.^76^ A total of 27 mice (11 intervention, 16 controls) were systemically treated with neurotrophin-3 and monitored for 2 weeks. Neurotrophin-3 treatment improved hearing thresholds with 10–20 dB depending on the stimulus and reduced neuronal loss in the cochlea by 30%. There was no improvement of clinical severity or histological brain damage.

### Paquinimod

Paquinimod is a quinolone derivative with immunomodulating properties. The proposed mechanism of action is through binding of the protein S100A9, a chemotactic inflammatory mediator involved in the recruitment of myeloid cells (Fig. 2.).^77,78^ Two studies evaluated paquinimod as adjunctive treatment, the first using eight treated mice and nine controls, the second nine treated mice and nine controls. Both studies showed that treatment decreased CSF leucocyte infiltration by half and an improvement of clinical scores of 60% 1 day after the start of treatment, although mortality was not improved.

### Effect of age on treatment

Age is an important prognostic factor in pneumococcal meningitis. Adjunctive dexamethasone treatment in infant rats was associated with reduced learning capacity, increased hippocampal apoptosis and increased mortality and decreased hippocampal neuronal regeneration.^79,80^ In adult rats, adjunctive dexamethasone treatment was associated with decreased memory impairment but increased oxidative stress.^81^ Adjunctive dexamethasone treatment in adult mice showed decreased mortality and slower increase in clinical scores.^50^ Daptomycin treatment was evaluated in infant rats and adult mice and improved hearing in both groups.^26,27,29^

## Discussion

The most promising adjunctive treatment in pneumococcal meningitis is blocking the complement system, more specifically C5 or its cleavage product C5a. In bacterial meningitis patients, genetic variation in complement genes and high levels of complement proteins in the CSF have been associated with poor outcomes, with the strongest associations found for complement C5.^82^ Variation in the C5 gene was associated with lower CSF leucocyte counts and unfavourable outcome in pneumococcal meningitis patients, and protein level of C5a in the CSF was associated with higher leucocytes counts, mortality, unfavourable outcome and delayed cerebral thrombosis.^49-84^ In mouse studies, animals deficient in C5a receptor had improved clinical scores and reduced inflammation.^49^ In this review, C5 antibody treatment was tested in three studies and improved clinical outcomes in all of them. Importantly, C5 antibodies had an additive effect in combination with dexamethasone, the current standard adjunctive treatment.^3,17,85^ The effect sizes were substantial, with a reduced mortality of ∼30% in multiple studies.^49,50^ Additionally, the quality of studies was above average as two of three studies were randomized and blinded.

When activated, complement C5 is cleaved into C5a, a potent anaphylatoxin, and C5b, the first component of the membrane attack complex (MAC) that kills pathogens.^86^ The anaphylatoxin C5a is the therapeutic target of interest as it strongly attracts leucocytes to the meninges and contributes to disease severity and poor clinical outcome.^82^ The MAC complex is not a modulator of the inflammatory response, but is necessary for bacterial killing, especially during meningococcal disease.^83^ The difficulty with targeting C5 in patients with meningitis is that the formation of both C5a and MAC is inhibited, and inhibiting the MAC might be harmful for patients infected with meningococci. A better strategy would be to specifically inhibit the C5a receptor (C5aR) or C5a, which *in vitro* prevented the harmful effects of C5a without inhibiting the MAC complex.^82,87,88^ Several drugs targeting complement have been approved for patients, such as the C5 inhibitors eculizumab and raviluzumab for the treatment of paroxysmal nocturnal haemoglobinuria, and the C5aR inhibitor avacopan used in anti-neutrophil cytoplasmic autoantibody (ANCA)-associated vasculitis. More complement inhibitors are under investigation, including the C5a inhibitor vilobelimab.^89,90^ Recent clinical trials showed that in addition to standard of care, vilobelimab improved survival of invasive mechanically ventilated patients with coronavirus disease 2019 (COVID-19).^91-93^ The blood–brain barrier poses another challenge to use of complement inhibitors, including monoclonal antibodies. Although the blood-brain barrier (BBB) is critical for brain homeostasis, it also prevents many drugs, including monoclonal antibodies, to enter the central nervous system, complicating the treatment of neurological disease. However, increased BBB permeability during inflammatory disease can allow antibodies to enter the CSF. Indeed, in patients with neuromyelitis optica spectrum disorder, an inflammatory disease of the brain, treatment with eculizumab reduced attack frequency, indicating that the antibodies can cross the BBB during inflammation. Since increased BBB permeability in bacterial meningitis is well documented, the penetration of drugs might be facilitated.

Another identified adjunctive treatment is daptomycin. Meningitis patients are typically treated with beta-lactam antibiotics that lyse bacteria, causing a quick release of bacterial components.^14^ Host cells recognize these bacterial components and activate the immune system, contributing to hyperinflammation characterized by an influx of leucocytes and the release of inflammatory mediators. Treatment with non-lytic antibiotics could decrease the release of bacterial components thereby limiting inflammation. Daptomycin is a non-lytic antibiotic that disrupts the bacterial membrane. In experimental meningitis, daptomycin treatment resulted in reduced cell wall lysis, lower concentrations of inflammatory markers and reduced brain damage when compared to treatment with ceftriaxone.^15,94,95^ In this review, daptomycin was well-evaluated, in five studies of which two were randomized blinded trials, and improved clinical parameters. Daptomycin alone reduced hearing loss and slightly ameliorated clinical scores, and in combination with C5 antibody, doxycycline or cipemastat, hearing, cognition or clinical severity score were improved. Because daptomycin is widely used for treatment of bacterial infections, it could be relatively easily adopted for treatment of meningitis. However, although pneumococcal strains are usually sensitive to treatment with daptomycin, treatment of pneumococcal pneumonia in patients with daptomycin was less effective than treatment with ceftriaxone.^96,97^ Additionally, daptomycin is not effective against Gram-negative bacteria and so will not be adequate for treatment of meningococcal meningitis. In the clinical setting, daptomycin cannot be given as sole treatment, but would have to be given adjunctive to standard antibiotic treatment, for example in combination with ceftriaxone. Other concerns regarding the adoption of daptomycin include the limited penetration into the CSF and adverse events associated with daptomycin use such as rhabdomyolysis and eosinophilic pneumonia.^98,99^ A recent literature review described 21 case reports of bacterial meningitis treated with daptomycin, although none of these were pneumococcal meningitis.^100^ Therapy was generally well tolerated, although there were two cases of daptomycin-induced asymptomatic myopathy. Currently, a phase 2 clinical trial testing adjunctive daptomycin in pneumococcal meningitis is underway, with an estimated completion date in 2024 (ClinicalTrials.gov identifier NCT03480191).

Doxycycline might also be a promising adjunctive therapy using non-lytic antibiotics. Also, a widely used drug it could quickly be applied in the clinical setting. In this review, treatment with doxycycline potently improved mortality and hearing in one large study, but results should be verified in well-designed experimental trials.

Several other potentially effective adjunctive treatments were identified. During pneumococcal meningitis, reactive oxygen and nitrogen species are produced in large amounts and are associated with clinical outcomes.^32,33^ Experimental models showed that oxidative stress in meningitis contributes to inflammation, neuronal cell death and cochlear damage.^34-38^ Adjunctive treatment with antioxidants was frequently evaluated, albeit only in small, non-randomized or unblinded studies. Results were variable, with some studies showing reduced inflammation or protection against cerebral or cochlear damage. Antioxidant treatment with NAC and MnTBAP improved hearing loss, but no other clinical parameters. As such, adjunctive antioxidant treatment may be beneficial in reducing hearing loss, but this needs to be confirmed in well-designed experimental trials. NAC would be the most attractive candidate for clinical trials, as it is already used for treatment of patients with acetaminophen toxicity, and has been tested in clinical trials for nephropathy and cystic fibrosis.^101-103^ However, there is reason to be cautious about the potential of antioxidant therapy. Although oxidative stress plays a role in many diseases including autoimmune disease, neurodegenerative disease and cancer, and preclinical trials showed therapeutic benefits in these diseases, the results of clinical trials have been disappointing. Reasons for this include that in some cases oxidative stress might be only a secondary contributor to disease pathology instead of the primary cause, the negligible effect of scavenging by most antioxidant drugs and the difficulty of achieving effective *in vivo* concentrations.^104,105^

HMGB1 inhibition with either Box A protein, ethyl pyruvate or HMGB1 antibodies improved clinical scores and reduced inflammation in this review. However, these drugs were only tested once and the experiments should be independently repeated. Treatment with HMGB1 antagonists was successful in preclinical models of many diseases including sepsis, arthritis or autoimmune carditis.^106^ Clinically only ethyl pyruvate has been tested in a trial with patients undergoing cardiac surgery, where it was generally well tolerated although it did not improve outcomes.^107^

MMP inhibitors were first evaluated for the treatment of cancer and chronic inflammatory diseases such as arthritis, but clinical trial results were disappointing, in part due to a low bioavailability and long-term adverse events such as myalgias resulting from unspecific MMP inhibition.^108,109^ The development of more specific MMP inhibitors and increased understanding of MMP biology led to a renewed interest in targeting the MMP pathway. In this review, MMP inhibition with BB-1101, GM6001 and cipemastat improved clinical scores or reduced cognitive deficits, although each drug was only investigated in one trial. Cipemastat might be the most promising MMP inhibitor, since this drug is a selective MMPs inhibitor, was evaluated in well-designed experimental trial and has already been tested in human patients with rheumatoid arthritis.^110^

Treatment with neurotrophins, especially with BDNF, has been extensively researched in neurodegenerative diseases such as Alzheimer, Parkinson and Huntington diseases.^111^ Decreased levels of BDNF were found in the brains of these patients and treatment with BNDF rescued neurons from cell death in animal studies.^111,112^ In this review, adjunctive treatment with neurotrophins might be protective against hearing loss, as treatment with BDNF and neurotrophin-3 reduced hearing impairment in one study each. However, delivery of neurotrophins is a challenge due to the short plasma half-life and limited diffusion of neurotrophins across the blood–brain barrier, consequently adjunctive BDNF was administered through a cannula implanted in the cerebral ventricles in included studies.^68,69,111,113,114^ Alternatively, neurotrophins might be delivered intranasally or production could indirectly be stimulated with exercise or drugs.^115^

Finally, paquinimod strongly reduced clinical severity, but only in two small unblinded trials and results need to be replicated. Clinically paquinimod has been evaluated in several trials investigating systemic lupus erythematosus (SLE), where it was well tolerated although dose-dependent adverse events were reported.^116,117^

To further improve the outcome of bacterial meningitis, a better understanding of the molecular and cellular mechanisms, of the involved signalling pathways and of the genes underlying the disease development and course will be helpful. The integration of basic fundamental research on genes and signalling pathways, use of genetically modified animal models (excluded in this review), use of gene editing techniques, patient data from the clinic, analysed data from reference laboratories, genome-wide association studies and knowledge about local administration of interventions might give us new insights and better understanding of the processes and lead us to better treatment strategies and interventions by well estimated and strongly motivated choices of adjuvant therapies.

Future laboratory animal experimental trials should adhere to international guidelines for good experimental procedures, laboratory practice and reporting, such as those described in Animal Research: Reporting of In Vivo Experiments (ARRIVE) or Planning Research and Experimental Procedures on Animals: Recommendations for Excellence (PREPARE), in order to be able to make firm, statistically significant, reproducible and reliable conclusions.^118,119^ Reporting of methods should be improved, for example with SYRCLE’s risk of bias tool. Recommendations on reporting of animal experiments advice that studies should justify sample sizes with a power calculation, describe relevant baseline characteristics of experimental groups, randomize animals to control and treatment groups, blind investigators for experimental groups during experiment and outcome assessment, randomize animals for outcome assessment, describe dropout of animals and randomize housing. Although in some countries, a power calculation and other SYRCLE criteria are prerequisites for animal experiment approval and, therefore, a lack of information on these topics does not imply that they were not conducted, these items should be reported in the manuscript. Finally, results from trials should be replicated, preferably in different experimental settings such as using a different animal species, pneumococcal serotype and animals of both sexes.

From experimental testing until implementation in clinical practice, promising adjunctive therapies have a long road to navigate as exemplified by the history of dexamethasone treatment in meningitis. Animal studies performed in the 1980s showed that corticosteroid treatment reduced CSF inflammation, brain oedema and improved outcomes, providing a rationale for giving corticosteroids to patients.^120,121^ Randomized clinical trials in patients investigating corticosteroids were already conducted in the 1960s in both children and adults, although with conflicting results.^122-126^ Finally, introduction into clinical practice was achieved after a European trial showed a beneficial effect of dexamethasone in all bacterial meningitis patients in 2002, with the largest effect found in pneumococcal meningitis.^17^

New experimental treatments should meet several requirements before progressing to a clinical trial, to maximize the chances that a beneficial effect will be found in a clinical setting. First, an experimental treatment should improve clinical outcomes and effect sizes should be large. Secondly, new adjunctive treatment should be effective when given in combination with adjunctive dexamethasone (which is the recommended treatment in developed regions), or when given alone, since in low-income countries regions adjunctive dexamethasone did not show benefits in trials and is not recommended.^16,17^ Additionally, treatment should not only be effective in pneumococcal meningitis, but also for other pathogens, or perhaps more important—not be harmful in patients with meningitis due to other pathogens. In clinical practice, antibiotic treatment in combination with dexamethasone treatment is most effective when given early, and the same time-dependent effect can be expected for a new experimental treatment.^17,127^ Finally, progression of a drug from the experimental to the clinical setting will be facilitated if the drug has already been tested in human patients or even approved for another indication. A challenge for clinical trials will be to include enough patients to be able to have enough power to detect a clinical effect. Bacterial meningitis occurs most frequently in developing countries and is rare in developed regions. Therefore, to be able to include enough patients, clinical trials should either be performed in developing countries or large multicentre trials in developed regions.

There are several limitations to this systematic review: the number of included papers and studies is low, the heterogeneity of the experiments regarding species, age, sex, experimental design and treatment is high, and the quality of study reporting is low.^25^ Evaluating the treatment effect of adjunctive treatment was complicated by study heterogeneity and it was not possible to use statistical methods to compare treatment effects or perform meta-analysis. Also, because of a lack of studies, it was not possible to determine the effect of age on treatment for most therapies. Furthermore, the studies included are limited to adjunctive treatments on wild type animals; studies on genetically modified animals are not included for translational reasons but might provide relevant information for the development of treatment options and therapies. Finally, in all included studies, pneumococcal meningitis was induced by direct inoculation of bacteria into the subarachnoid space. However, in patients, pneumococci generally invade the subarachnoid space from the bloodstream, either through the blood–brain barrier or the blood–CSF barrier in the choroid plexus.^8,128^ The meningitis model with intracisternal inoculation is suited for investigating meningitis once infection is established, but not for evaluating the events leading up to CSF infection. To study the natural route of infection, models where meningitis is induced through intranasal, haematological or peritoneal routes might be more adequate^129^ However, the drawback of these indirect infection models is that more than half of the animals will not develop meningitis, even in animals who develop severe sepsis.^130,131^ For studies investigating treatments, direct inoculation into the subarachnoid space is regarded preferable with reduction of the number of animals needed per experiment and reproducible results.^129,132^

In conclusion, we identified 54 adjunctive treatment regimens that were tested in an experimental animal model of pneumococcal meningitis. Of these 54 regimens, 24 (44%) reduced disease severity, reduced hearing loss or cognitive impairment, but few were tested in well-designed studies and independently validated. The strongest evidence exists for adjunctive therapy with C5 antibodies or daptomycin. Many studies included in our systematic review were hampered by methodological flaws. Future studies should adhere to current guidelines for animal research including those regarding randomization, blinding and the reporting of methodology.

## Supplementary Material

fcae131_Supplementary_Data

## Data Availability

All data generated or analysed during this study are included in this published article (and its supplementary files) or available on request from the corresponding author.
