# Supplementary material for: Adjunctive treatments for pneumococcal meningitis: a systematic review of experimental animal models
Source: Brain Commun. 2024 Apr 12;6(3):fcae131. doi: 10.1093/braincomms/fcae131 (PMC11069119; doi:10.1093/braincomms/fcae131)
Supplement: fcae131_Supplementary_Data [file fcae131_supplementary_data.zip › Supplementary File 1.pdf]

Supplementary file 1. Search strategy

Search strategy per database.
Date of search: Feb 16, 2023.

1. **Medline**

**Ovid MEDLINE(R) ALL <1946 to February 16, 2023>**	
#	**Searches**
1	exp Animal Experimentation/
2	exp Models, Animal/
3	Animals/
4	exp Mice/
5	exp Rats/
6	exp Rabbits/
7	exp Zebrafish/
8	exp Swine/
9	exp Guinea Pigs/ or exp Dogs/
10	(mice or mouse or pig or pigs or rat or rats or rabbit* or swine or zebrafish or rodent or rodents or danio or dog or dogs or animal-model* or (animal adj2 experiment*) or (strain adj "129") or c57bl or balb-c or c3h or wist or wistar).ti,ab,kf.
11	1 or 2 or 3 or 4 or 5 or 6 or 7 or 8 or 9 or 10
12	exp Meningitis, Pneumococcal/
13	meningitis.ti,ab.
14	Meningitis/
15	exp Streptococcus pneumoniae/ or exp Pneumococcal Infections/
16	(pneumoniae or pneumococcal).ti,ab,kf.
17	15 or 16
18	13 or 14
19	17 and 18
20	12 or 19
21	(experimental adj3 meningitis).ti,ab.
22	11 and 20
23	21 or 22

2. **Embase**

#	**Query**
#1	'animal use'/exp OR 'male animal'/exp OR 'juvenile animal'/exp OR 'transgenic animal'/exp OR 'experimental animal'/exp OR 'female animal'/exp OR 'animal'/de OR 'animal experiment'/exp OR 'animal model'/exp OR 'mouse'/exp OR 'rat'/exp OR 'leporidae'/exp OR 'zebra fish'/exp OR 'pig'/exp OR 'guinea pig'/exp OR 'dog'/exp OR mice:ti,ab,kw OR mouse:ti,ab,kw OR pig:ti,ab,kw OR pigs:ti,ab,kw OR rat:ti,ab,kw OR rats:ti,ab,kw OR rabbit*:ti,ab,kw OR swine:ti,ab,kw OR zebrafish:ti,ab,kw OR rodent:ti,ab,kw OR rodents:ti,ab,kw OR danio:ti,ab,kw OR dog:ti,ab,kw OR dogs:ti,ab,kw OR 'animal model*':ti,ab,kw OR ((animal NEAR/2 experiment*):ti,ab,kw) OR ((strain NEXT/1 '129'):ti,ab,kw) OR c57bl:ti,ab,kw OR 'balb c':ti,ab,kw OR c3h:ti,ab,kw OR wist:ti,ab,kw OR wistar:ti,ab,kw
#2	'pneumococcal meningitis'/de OR (('meningitis'/de OR meningitis:ti,ab) AND ('streptococcus pneumoniae'/exp OR 'pneumococcal infection'/exp OR pneumoniae:ti,ab,kw OR pneumococcal:ti,ab,kw))

#3	#1 AND #2
#4	((experimental NEAR/3 meningitis):ti,ab,kw) OR 'experimental pneumococcal meningitis'/de OR 'experimental meningitis'/de
#5	#3 OR #4
#6	#5 AND [embase]/lim

3. Scopus

#	**Query**
1	TITLE-ABS-KEY (((mice OR mouse OR pig OR pigs OR rat OR rats OR rabbit* OR swine OR zebrafish OR rodent OR rodents OR danio OR dog OR dogs OR animal-model* OR (animal W/2 experiment*) OR (strain W/1 129) OR c57bl OR balb-c OR c3h OR wist OR wistar) AND (meningitis AND (pneumoniae OR pneumococcal OR streptoc*))) OR (experimental W/1 meningitis))
