# Supplementary material for: Adjunctive treatments for pneumococcal meningitis: a systematic review of experimental animal models
Source: Brain Commun. 2024 Apr 12;6(3):fcae131. doi: 10.1093/braincomms/fcae131 (PMC11069119; doi:10.1093/braincomms/fcae131)
Supplement: fcae131_Supplementary_Data [file fcae131_supplementary_data.zip › Supplementary_tables.pdf]

Supplementary Table 1. Studies included in the systematic review

Study	Article	Authors	Journal	Year
Addison	Cochlear preservation after meningitis: an animal model confirmation of adjunctive steroid therapy	Addison, J. and Kim, H. H. and Richter, C. P.	Laryngoscope	2006
Bally	Inhibition of Hippocampal Regeneration by Adjuvant Dexamethasone in Experimental Infant Rat Pneumococcal Meningitis	Bally, L. and Grandgirard, D. and Leib, S. L.	Antimicrobial Agents & Chemotherapy	2016
Barichello (1)	Dexamethasone treatment reverses cognitive impairment but increases brain oxidative stress in rats submitted to pneumococcal meningitis	Barichello, T. and Santos, A. L. and Silvestre, C. and Generoso, J. S. and Cipriano, A. L. and Petronilho, F. and Dal-Pizzol, F. and Comim, C. M. and Quevedo, J.	Oxidative medicine & cellular longevity	2011
Barichello (2)	Effect of new compound with antioxidant potential in the energy metabolism of adults rats after pneumococcal meningitis	Barichello, T. and Savi, G. D. and Panatto, A. P. and Generoso, J. S. and Cipriano, A. L. and Rezin, G. T. and Dal-Pizzol, F. and Streck, E. L. and Petronilho, F. and Vidal, L. and Geffard, M.	American Journal of Pharmacology and Toxicology	2012
Barichello (3)	Folic acid prevented cognitive impairment in experimental pneumococcal meningitis	Barichello, T. and Generoso, J. S. and Simoes, L. R. and Steckert, A. V. and Moreira, A. P. and Dominguini, D. and Ferrari, P. and Gubert, C. and Kapczinski, F. and Jornada, L. K. and Danielski, L. G. and Petronilho, F. and Budni, J. and Quevedo, J.	Journal of Neural Transmission	2015
Bass	A novel nonpsychotropic cannabinoid, HU-211, in the treatment of experimental pneumococcal meningitis	Bass, R. and Engelhard, D. and Trembovler, V. and Shohami, E.	Journal of Infectious Diseases	1996
Bhatt	The impact of dexamethasone on hearing loss in experimental pneumococcal meningitis	Bhatt, S. M. and Cabellos, C. and Nadol, J. B., Jr. and Halpin, C. and Lauretano, A. and Xu, W. Z. and Tuomanen, E.	Pediatric Infectious Disease Journal	1995
Blaser (1)	Adjuvant glycerol is not beneficial in experimental pneumococcal meningitis	Blaser, C. and Klein, M. and Grandgirard, D. and Wittwer, M. and Peltola, H. and Weigand, M. and Koedel, U. and Leib, S. L.	BMC Infectious Diseases	2009
Blaser (2)	Adjunctive dexamethasone affects the expression of genes related to inflammation, neurogenesis and apoptosis in infant rat pneumococcal meningitis	Blaser, C. and Wittwer, M. and Grandgirard, D. and Leib, S. L.	PLoS ONE [Electronic Resource]	2011
Brandt (1)	Attenuation of the bacterial load in blood by pretreatment with granulocyte-colony-stimulating factor protects rats from fatal outcome and brain damage during Streptococcus pneumoniae meningitis	Brandt, C. T. and Lundgren, J. D. and Lund, S. P. and Frimodt-Moller, N. and Christensen, T. and Benfield, T. and Espersen, F. and Hougaard, D. M. and Ostergaard, C.	Infection & Immunity	2004
Brandt (2)	Evaluation of anti-pneumococcal capsular antibodies as adjunctive therapy in experimental pneumococcal meningitis	Brandt, C. T. and Frimodt-Moller, N. and Lundgren, J. D. and Pedersen, M. and Skovsted, I. C. and Rowland, I. J. and Ostergaard, C.	Journal of Antimicrobial Chemotherapy	2006
Braun	Neuroprotection by a caspase inhibitor in acute bacterial meningitis	Braun, J. S. and Novak, R. and Herzog, K. H. and Bodner, S. M. and Cleveland, J. L. and Tuomanen, E. I.	Nature Medicine	1999
Cabellos	Influence of dexamethasone on efficacy of ceftriaxone and vancomycin therapy in experimental pneumococcal meningitis	Cabellos, C. and Martinez-Lacasa, J. and Martos, A. and Tubau, F. and Fernandez, A. and Viladrich, P. F. and Gudiol, F.	Antimicrobial Agents & Chemotherapy	1995
Coimbra	Limited efficacy of adjuvant therapy with dexamethasone in preventing hearing loss due to experimental pneumococcal meningitis in the infant rat	Coimbra, R. S. and Loquet, G. and Leib, S. L.	Pediatric Research	2007
Demel	Reduced spiral ganglion neuronal loss by adjunctive neurotrophin-3 in experimental pneumococcal meningitis	Demel, C. and Hoegen, T. and Giese, A. and Angele, B. and Pfister, H. and Koedel, U. and Klein, M.	Journal of Neuroinflammation	2011
Demirbas	Early dexamethasone treatment enhances hearing preservation after pneumococcal meningitis: An animal study	Demirbas, O. and Basar, F. and Atmaca, S. and Tekat, A.	Journal of International Advanced Otology	2009
Erni	Anti-inflammatory and Oto-Protective Effect of the Small Heat Shock Protein Alpha B-Crystallin (HspB5) in Experimental Pneumococcal Meningitis	Erni, S. T. and Fernandes, G. and Buri, M. and Perny, M. and Rutten, R. J. and van Noort, J. M. and Senn, P. and Grandgirard, D. and Roccio, M. and Leib, S. L.	Frontiers in neurology [electronic resource].	2019

Ertunc	A comparison of the effectiveness of erythropoietin and dexamethasone therapy in streptococcus pneumoniae induced meningitis in rabbits	Ertunc, N. C. and Kamasak, T. and Buruk, K. and Bayramoglu, G. and Alver, A. and Mentese, A. and Canpolat, S. and Erduran, E. and Cansu, A.	Current Pediatric Research	2017
Ge	The effects of superoxide dismutase in gerbils with bacterial meningitis	Ge, N. N. and Brodie, S. A. and Tinling, S. P. and Brodie, H. A.	Otolaryngology - Head & Neck Surgery	2004
Gerber	Intrathecal treatment with the anti-phosphorylcholine monoclonal antibody TEPC-15 decreases neuronal damage in experimental pneumococcal meningitis	Gerber, J. and Redlich, S. and Ribes, S. and Tauber, S. C. and Schmidt, H. and Nau, R.	Chemotherapy	2012
Grandgirard	Adjunctive daptomycin attenuates brain damage and hearing loss more efficiently than rifampin in infant rat pneumococcal meningitis	Grandgirard, D. and Burri, M. and Agyeman, P. and Leib, S. L.	Antimicrobial Agents & Chemotherapy	2012
Granert (1)	The polysaccharide fucoidin inhibits the antibiotic-induced inflammatory cascade in experimental pneumococcal meningitis	Granert, C. and Raud, J. and Lindquist, L.	Clinical & Diagnostic Laboratory Immunology	1998
Granert (2)	Effects of polysaccharide fucoidin on cerebrospinal fluid interleukin-1 and tumor necrosis factor alpha in pneumococcal meningitis in the rabbit	Granert, C. and Raud, J. and Waage, A. and Lindquist, L.	Infection & Immunity	1999
Hogen	Adjunctive N-acetyl-L-cysteine in treatment of murine pneumococcal meningitis	Hogen, T. and Demel, C. and Giese, A. and Angele, B. and Pfister, H. W. and Koedel, U. and Klein, M.	Antimicrobial Agents & Chemotherapy	2013
Hohne	High mobility group box 1 prolongs inflammation and worsens disease in pneumococcal meningitis	Hohne, C. and Wenzel, M. and Angele, B. and Hammerschmidt, S. and Hacker, H. and Klein, M. and Bierhaus, A. and Sperandio, M. and Pfister, H. W. and Koedel, U.	Brain	2013
Kasanmoen. (1)	Adjuvant treatment with dexamethasone plus anti-C5 antibodies improves outcome of experimental pneumococcal meningitis: a randomized controlled trial	Kasanmoentalib, E. S. and Valls Seron, M. and Morgan, B. P. and Brouwer, M. C. and van de Beek, D.	Journal of Neuroinflammation	2015
Kasanmoen. (2)	Mannose-binding lectin-associated serine protease 2 (MASP-2) contributes to poor disease outcome in humans and mice with pneumococcal meningitis	Kasanmoentalib, E. S. and Valls Seron, M. and Ferwerda, B. and Tanck, M. W. and Zwinderman, A. H. and Baas, F. and van der Ende, A. and Brouwer, M. C. and van de Beek, D.	Journal of Neuroinflammation	2017
Kasanmoen. (3)	Complement factor H contributes to mortality in humans and mice with bacterial meningitis	Kasanmoentalib, E. S. and Valls Seron, M. and Engelen-Lee, J. Y. and Tanck, M. W. and Pouw, R. B. and van Mierlo, G. and Wouters, D. and Pickering, M. C. and van der Ende, A. and Kuijpers, T. W. and Brouwer, M. C. and van de Beek, D.	Journal of Neuroinflammation	2019
Kastenbauer (1)	Reactive nitrogen species contribute to blood-labyrinth barrier disruption in suppurative labyrinthitis complicating experimental pneumococcal meningitis in the rat	Kastenbauer, S. and Klein, M. and Koedel, U. and Pfister, H. W.	Brain Research	2001
Kastenbauer (2)	Pneumococcal meningitis in the rat: evaluation of peroxynitrite scavengers for adjunctive therapy	Kastenbauer, S. and Koedel, U. and Becker, B. F. and Pfister, H. W.	European Journal of Pharmacology	2002
Kim	Otoprotective effects of dexamethasone in the management of pneumococcal meningitis: an animal study	Kim, H. H. and Addison, J. and Suh, E. and Trune, D. R. and Richter, C. P.	Laryngoscope	2007
Kirschnek	Apoptosis is essential for neutrophil functional shutdown and determines tissue damage in experimental pneumococcal meningitis	Kirschnek, S. and Frankenberg, T. and Koedel, U. and Obermaier, B. and Hacker, H. and Paul, R. and Hacker, G.	European Journal of Immunology	2009
Klein (1)	Meningitis-associated hearing loss: protection by adjunctive antioxidant therapy	Klein, M. and Koedel, U. and Pfister, H. W. and Kastenbauer, S.	Annals of Neurology	2003
Klein (2)	Adjuvant non-bacteriolytic and anti-inflammatory combination therapy in pneumococcal meningitis: an investigation in a mouse model	Klein, M. and Hohne, C. and Angele, B. and Hogen, T. and Pfister, H. W. and Tufekci, H. and Koedel, U.	Clinical Microbiology & Infection	2019
Le	The CCR5 antagonist maraviroc exerts limited neuroprotection without improving neurofunctional outcome in experimental pneumococcal meningitis	Le, N. D. and Steinfort, M. and Grandgirard, D. and Maleska, A. and Leppert, D. and Kuhle, J. and Leib, S. L.	Scientific Reports	2022

Leib (1)	Inhibition of matrix metalloproteinases and tumour necrosis factor alpha converting enzyme as adjuvant therapy in pneumococcal meningitis	Leib, S. L. and Clements, J. M. and Lindberg, R. L. and Heimgartner, C. and Loeffler, J. M. and Pfister, L. A. and Tauber, M. G. and Leppert, D.	Brain	2001
Leib (2)	Dexamethasone aggravates hippocampal apoptosis and learning deficiency in pneumococcal meningitis in infant rats	Leib, S. L. and Heimgartner, C. and Bifrare, Y. D. and Loeffler, J. M. and Taauber, M. G.	Pediatric Research	2003
Li	Neuroprotective effects of brain-derived neurotrophic factor (BDNF) on hearing in experimental pneumococcal meningitis	Li, L. and Shui, Q. X. and Li, X.	Journal of Child Neurology	2005
Lui	Efficacy of GM6001 as an adjuvant to ceftriaxone in a neonatal rat model of Streptococcus pneumoniae meningitis	Liu, X. and Han, Q.	Acta Neurobiologiae Experimentalis	2014
Lutsar	Factors influencing the anti-inflammatory effect of dexamethasone therapy in experimental pneumococcal meningitis	Lutsar, I. and Friedland, I. R. and Jafri, H. S. and Wubbel, L. and Ahmed, A. and Trujillo, M. and McCoig, C. C. and McCracken, G. H., Jr.	Journal of Antimicrobial Chemotherapy	2003
Masouris	Inhibition of DAMP signaling as an effective adjunctive treatment strategy in pneumococcal meningitis	Masouris, I. and Klein, M. and Dyckhoff, S. and Angele, B. and Pfister, H. W. and Koedel, U.	Journal of Neuroinflammation	2017
Meli	Doxycycline reduces mortality and injury to the brain and cochlea in experimental pneumococcal meningitis	Meli, D. N. and Coimbra, R. S. and Erhart, D. G. and Loquet, G. and Bellac, C. L. and Tauber, M. G. and Neumann, U. and Leib, S. L.	Infection & Immunity	2006
Muri (1)	Combined effect of non-bacteriolytic antibiotic and inhibition of matrix metalloproteinases prevents brain injury and preserves learning, memory and hearing function in experimental paediatric pneumococcal meningitis	Muri, L. and Grandgirard, D. and Buri, M. and Perny, M. and Leib, S. L.	Journal of Neuroinflammation	2018
Muri (2)	Combining Ceftriaxone with Doxycycline and Daptomycin Reduces Mortality, Neuroinflammation, Brain Damage, and Hearing Loss in Infant Rat Pneumococcal Meningitis	Muri, L. and Perny, M. and Zemp, J. and Grandgirard, D. and Leib, S. L.	Antimicrobial Agents & Chemotherapy	2019
Muri (3)	Metformin mediates neuroprotection and attenuates hearing loss in experimental pneumococcal meningitis	Muri, L. and Le, N. D. and Zemp, J. and Grandgirard, D. and Leib, S. L.	Journal of Neuroinflammation	2019
Pan	Adjuvant Cannabinoid Receptor Type 2 Agonist Modulates the Polarization of Microglia Towards a Non-Inflammatory Phenotype in Experimental Pneumococcal Meningitis	Pan, S. D. and Grandgirard, D. and Leib, S. L.	Frontiers in Cellular & Infection Microbiology	2020
Rappaport	Prevention of hearing loss in experimental pneumococcal meningitis by administration of dexamethasone and ketorolac	Rappaport, J. M. and Bhatt, S. M. and Burkard, R. F. and Merchant, S. N. and Nadol, J. B., Jr.	Journal of Infectious Diseases	1999
Song	The responsiveness of TrkB to exogenous BDNF in frontal cortex during antibiotic treatment of Streptococcus pneumoniae meningitis	Song, X. and Lian, D. and He, D. and Sun, J. and Zhu, M. and Li, L.	Neurological Sciences	2014
Spreer (1)	Antiinflammatory but no neuroprotective effects of melatonin under clinical treatment conditions in rabbit models of bacterial meningitis	Spreer, A. and Gerber, J. and Baake, D. and Hanssen, M. and Huether, G. and Nau, R.	Journal of Neuroscience Research	2006
Spreer (2)	Short-term rifampicin pretreatment reduces inflammation and neuronal cell death in a rabbit model of bacterial meningitis	Spreer, A. and Lugert, R. and Stoltefaut, V. and Hoecht, A. and Eiffert, H. and Nau, R.	Critical Care Medicine	2009
Wache	Myeloid-related protein 14 promotes inflammation and injury in meningitis	Wache, C. and Klein, M. and Ostergaard, C. and Angele, B. and Hacker, H. and Pfister, H. W. and Pruenster, M. and Sperandio, M. and Leanderson, T. and Roth, J. and Vogl, T. and Koedel, U.	Journal of Infectious Diseases	2015
Woehrl (1)	CXCL16 contributes to neutrophil recruitment to cerebrospinal fluid in pneumococcal meningitis	Woehrl, B. and Klein, M. and Rupprecht, T. and Schmetzer, H. and Angele, B. and Hacker, H. and Hacker, G. and Pfister, H. W. and Koedel, U.	Journal of Infectious Diseases	2010
Woehrl (2)	Complement component 5 contributes to poor disease outcome in humans and mice with pneumococcal meningitis	Woehrl, B. and Brouwer, M. C. and Murr, C. and Heckenberg, S. G. and Baas, F. and Pfister, H. W. and Zwinderman, A. H. and Morgan, B. P. and Barnum, S. R. and van der Ende, A. and Koedel, U. and van de Beek, D.	Journal of Clinical Investigation	2011
Worsoe (1)	Intratympanic steroid prevents long-term spiral ganglion neuron loss in experimental meningitis	Worsoe, L. and Brandt, C. T. and Lund, S. P. and Ostergaard, C. and Thomsen, J. and Caye-Thomasen, P.	Otology & Neurotology	2010

Worsoe (2)	Systemic steroid reduces long-term hearing loss in experimental pneumococcal meningitis	Worsoe, L. and Brandt, C. T. and Lund, S. P. and Ostergaard, C. and Thomsen, J. and Caye-Thomasen, P.	Laryngoscope	2010
Zhang	Regulation of the p75 neurotrophin receptor attenuates neuroinflammation and stimulates hippocampal neurogenesis in experimental Streptococcus pneumoniae meningitis	Zhang, D. and Zhao, S. and Zhang, Z. and Xu, D. and Lian, D. and Wu, J. and He, D. and Sun, K. and Li, L.	Journal of Neuroinflammation	2021
Zysk (1)	Anti-inflammatory treatment influences neuronal apoptotic cell death in the dentate gyrus in experimental pneumococcal meningitis	Zysk, G. and Bruck, W. and Gerber, J. and Bruck, Y. and Prange, H. W. and Nau, R.	Journal of Neuropathology & Experimental Neurology	1996
Zysk (2)	Limited efficacy of pentoxifylline as anti-inflammatory agent in experimental pneumococcal meningitis	G Zysk, W Brück, F R Fischer, M Mäder, P Rieckmann, R Nau	Clinical Experimental Immunology	1997

Supplementary table 2. Study characteristics

**Study**	**Experimental animal**	**Sex**	**Age**	**Number of animals**	**Serotype**	**Adjunctive drug(s)**	**Start adjunctive therapy**	**Antibiotic therapy**
Addison	gerbil	both	adult	12	N/A	dexamethason	12 hpi	penicillin G
Bally	rat	both	infant	90	3	dexamethason	18 hpi	ceftriaxone
Barichello (1)	rat	male	adult	20	N/A	dexamethason	16 hpi	ceftriaxone
Barichello (2)	rat	male	NA	10-14	3	P0801	16 hpi	ceftriaxone
Barichello (3)	rat	male	adult	30	3	folic acid	18 hpi	ceftriaxone
Bass	rat	male	NA	50	14	HU-211	18 hpi	ceftriaxone
Bhatt	rabbit	female	NA	10	3	dexamethason	12 hpi	ceftriaxone
Blaser (1)	rat, mouse	N/A	infant	50	2, 3	glycerol	18 hpi	ceftriaxone
Blaser (2)	rat	N/A	infant	32	3	dexamethason	18 hpi	ceftriaxone
Brandt (1)	rat	male	adult	48	3	G-CSF	28 hpi	ceftriaxone
Brandt (2)	rat	male	adult	50	3	serotype specific antiserum	26 hpi	ceftriaxone
Braun	rabbit	male	NA	9	2	z-VAD-fmk	8 hpi	ceftriaxone
Cabellos	rabbit	female	NA	16	3	dexamethason	18 hpi	ceftriaxone
Coimbra	rat	N/A	infant	36	N/A	dexamethason	18 hpi	ceftriaxone
Demel	mouse	N/A	adult	39	2	neurotrophin-3, dexamethason	18 hpi	ceftriaxone
Demirbas	rat	male	NA	20	3	dexamethason	18 hpi	ceftriaxone
Erni	rat	N/A	infant	~20	3	HspB5	18 hpi	ceftriaxone
Ertunc	rabbit	female	NA	14	N/A	erythropoietin (EPO)	12 hpi	ceftriaxone
Ge	gerbil	male	adult	30	3	superoxide dismutase (SOD)	24 hpi	penicillin G
Gerber	rabbit	N/A	NA	21	3	phosphorylcholine mouse IgA antibody	12 hpi	ceftriaxone
Grandgirard	rat	N/A	infant	112	3	rifampicine, daptomycin	18 hpi	ceftriaxone
Granert (1)	rabbit	female	NA	30	3	fucoidin	16 hpi	ampicillin
Granert (2)	rabbit	female	NA	10	3	fucoidin	16 hpi	ampicillin
Hogen	mouse	male	NA	26	2	N-Acetyl-L-Cysteine (NAC)	18 hpi	ceftriaxone
Hohne	mouse	male	adult	36	2	ethyl pyruvate (REPS), Box A protein	21 hpi	ceftriaxone
Kasanmoen. (1)	mouse	male	adult	96	3	C5 antibody, dexamethason	20 hpi	ceftriaxone
Kasanmoen. (2)	mouse	N/A	adult	84	3	MASP-2 antibodies	20 hpi	ceftriaxone
Kasanmoen. (3)	mouse	N/A	adult	67	3	factor H	16 hpi	ceftriaxone
Kastenbauer (1)	rat	male	adult	15	3	uric acid, MnTBAP	20 hpi	ceftriaxone
Kastenbauer (2)	rat	male	adult	39	4	uric acid, MnTBAP, ascorbate	20 hpi	ceftriaxone
Kim	gerbil	N/A	adult	8	3	dexamethason	12 hpi	penicillin G

Kirschnek	mouse	male	NA	42	2	roscovitine	18 hpi	ceftriaxone
Klein (1)	rat	male	adult	17	3	N-Acetyl-L-Cysteine (NAC), MnTBAP	18 hpi	ceftriaxone
Klein (2)	mouse	male	adult	59	2	C5 antibody, daptomycin, IL-1 antibody, roscovitine, dexamethasone	21 hpi	ceftriaxone
Le	rat	both	infant	84	3	Maraviroc	18 hpi	ceftriaxone
Leib (1)	rat	N/A	infant	104	3	BB-1101	18 hpi	ceftriaxone
Leib (2)	rat	N/A	infant	114	N/A	dexamethason	18 hpi	ceftriaxone
Li	rat	N/A	infant	16	3	BDNF	24 hpi	ceftriaxone
Lui	rat	N/A	infant	48	3	GM6001	24 hpi	ceftriaxone
Lutsar	rabbit	N/A	NA	24-26	N/A	dexamethason	24 hpi	ampicillin
Masouris	mouse	male	adult	57	2	paquinimod, daptomycin, HMGB1, dexamethason	21 hpi	ceftriaxone
Meli	rat	N/A	infant	180	N/A	doxycyclin	18 hpi	ceftriaxone
Muri (1)	rat	N/A	infant	156	3	cipemastat, daptomycin	18 hpi	ceftriaxone
Muri (2)	rat	N/A	infant	144	3	daptomycin, doxycyclin	18 hpi	ceftriaxone
Muri (3)	rat	both	infant	140	3	metformin	18 hpi	ceftriaxone
Pan	rat	both	infant	70	3	JWH-133	18 hpi	ceftriaxone
Rappaport	rabbit	female	NA	18	3	ketorolac, dexamethason	18 hpi	ampicillin
Song	rat	N/A	infant	32	3	BDNF	24 hpi	ceftriaxone
Spreer (1)	rabbit	N/A	NA	20	3	melatonin	12 hpi	ceftriaxone
Spreer (2)	rabbit	N/A	NA	24	3	rifampicine	12 hpi	ceftriaxone
Wache	mouse	male	adult	18	2	paquinimod	24 hpi	ceftriaxone
Woehrl (1)	mouse	N/A	adult	22	2	CXCL16 antibody	24 hpi	ceftriaxone
Woehrl (2)	mouse	male	NA	49	2	C5 antibody, dexamethason, TRL2 antibody, TLR4 antibody	24 hpi	ceftriaxone
Worsoe (1)	rat	male	adult	39	3	betamethason	21 hpi	ceftriaxone
Worsoe (2)	rat	male	adult	26	3	betamethason	21 hpi	ceftriaxone
Zhang	rat	N/A	infant	28	3	LM11A-32	24 hpi	ceftriaxone
Zysk (1)	rabbit	N/A	NA	20	3	CD18 antibody, dexamethason	16 hpi	ceftriaxone
Zysk (2)	rabbit	N/A	NA	19	3	pentoxifylline	16 hpi	ceftriaxone

Study characteristics of all included studies. Study: study name, Experimental animal: experimental animals used, Sex: sex of experimental animal, Age: age of experimental animal, Serotype: serotype of pneumococcal strain, Adjunctive drugs(s): adjunctive drug(s) used as therapy, Start adjunctive therapy: start of adjunctive therapy in hours after inoculation (hpi), Antibiotics: antibiotic drugs given with adjunctive treatment.
BDNF = brain-derived neurotrophic factor, C5 = complement component 5, CXCL16 = Chemokine (C-X-C motif) ligand 16, HMGB1 = High mobility group box 1 protein, HspB5 = small heat shock protein alpha B-crystallin, JWH-133 = Dimethylbutyl-deoxy-Delta-8-THC, MASP-2 = mannose-binding protein-associated serine protease 2, TLR = toll-like receptor,

Supplementary Table 3. Risk of bias assessment

Study	1. sequence generation	2. baseline characteristics	3. allocation concealment	4. random housing	5. blinding experimental groups	6. random outcome assessment	7. blinding outcome assessors	8. incomplete outcome data	9. selective outcome reporting
Addison	Y	U	U	U	U	Y	U	U	U
Bally	Y	U	U	U	U	Y	Y	Y	U
Barichello (1)	Y	U	U	U	U	U	U	U	U
Barichello (2)	U	U	U	U	U	Y	U	U	U
Barichello (3)	Y	Y	U	U	U	U	Y	Y	U
Bass	Y	Y	U	U	U	U	U	Y	U
Bhatt	Y	Y	U	U	U	U	U	Y	U
Blaser (1)	Y	Y	U	U	U	U	U	U	U
Blaser (2)	Y	Y	U	U	U	Y	U	Y	U
Brandt (1)	Y	U	U	U	U	Y	Y	Y	U
Brandt (2)	Y	Y	U	U	Y	Y	Y	Y	U
Braun	U	U	U	U	U	U	U	U	U
Cabellos	U	U	U	U	U	Y	U	U	U
Coimbra	Y	Y	U	U	U	U	Y	Y	U
Demel	U	Y	U	U	Y	Y	Y	Y	U
Demirbas	Y	Y	U	U	U	U	Y	Y	U
Erni	Y	Y	U	U	Y	Y	Y	U	U
Ertunc	U	Y	U	U	N	U	U	Y	U
Ge	U	Y	U	U	U	U	U	Y	U
Gerber	U	Y	U	U	U	Y	Y	U	U
Grandgirard	Y	U	U	U	U	Y	U	U	U
Granert (1)	U	Y	U	U	U	U	U	U	U
Granert (2)	U	U	U	U	U	Y	U	Y	U
Hogen	U	Y	U	U	Y	U	Y	U	U
Hohne	U	Y	U	U	U	Y	U	Y	U
Kasanmoen. (1)	Y	U	U	U	Y	Y	Y	Y	U
Kasanmoen. (2)	Y	Y	U	U	Y	Y	Y	Y	U
Kasanmoen. (3)	Y	Y	U	U	Y	Y	Y	Y	U
Kastenbauer (1)	U	U	U	U	U	Y	Y	U	U
Kastenbauer (2)	U	U	U	U	U	Y	U	U	U
Kim	Y	U	U	U	U	U	U	U	U
Kirschnek	U	Y	U	U	U	U	U	U	U
Klein (1)	Y	Y	U	U	U	U	Y	Y	U
Klein (2)	Y	Y	U	U	Y	Y	Y	U	U

Le	Y	Y	U	U	Y	Y	Y	U	U
Leib (1)	Y	U	U	U	U	U	Y	Y	U
Leib (2)	Y	U	U	U	U	U	Y	Y	U
Li	Y	Y	U	U	U	U	U	Y	U
Lui	Y	U	U	U	U	U	U	U	U
Lutsar	U	U	U	U	U	Y	U	U	U
Masouris	Y	Y	U	U	U	Y	Y	Y	U
Meli	Y	U	U	U	U	Y	Y	Y	U
Muri (1)	Y	Y	U	Y	U	Y	Y	U	U
Muri (2)	Y	Y	U	U	U	U	Y	Y	U
Muri (3)	Y	Y	U	U	Y	U	Y	Y	U
Pan	Y	Y	U	U	U	Y	U	U	U
Rappaport	Y	Y	U	U	Y	Y	Y	Y	U
Song	Y	Y	U	U	U	Y	Y	Y	U
Spreer (1)	Y	Y	U	U	U	U	Y	Y	U
Spreer (2)	Y	Y	U	U	U	U	Y	Y	U
Wache	U	Y	U	U	U	Y	U	U	U
Woehrl (1)	U	Y	U	U	U	U	U	U	U
Woehrl (2)	U	Y	U	U	U	U	U	U	U
Worsoe (1)	Y	Y	U	U	U	Y	Y	Y	U
Worsoe (2)	Y	Y	U	U	U	Y	Y	Y	U
Zhang	Y	U	U	U	U	Y	Y	U	U
Zysk (1)	U	Y	U	U	U	U	Y	U	U
Zysk (2)	Y	Y	U	U	U	U	U	U	U

Risk of bias assessment performed with the tool developed by the Systematic Review Centre for Laboratory animal Experimentation. Y = yes, U = unclear, N = no.

Supplementary Table 4. Adjunctive treatments and their effects in experimental pneumococcal meningitis

**Treatment**	**Study**	**Disease severity**		**Hearing loss**		**Cognitive impairment**		**Inflammation**		**Brain injury**		**Bacterial load**	
		**Parameter**	**Effect**	**Parameter**	**Effect**	**Parameter**	**Effect**	**Parameter**	**Effect**	**Parameter**	**Effect**	**Parameter**	**Effect**
**Complement**													
C5 ab	Woehrl (2)	mortality	↓							histopathology	↓		
	Kasanmoen. (1)	mortality<br>clinical score	↓<br>↓										
C5 ab + DEX	Kasanmoen. (1)	mortality<br>clinical score	↓<br>↓										
C5 ab + DAP	Klein (2)*	clinical score	↓	ABR	↓	explorative activity<br>T-maze	↓	CSF leukocytes	↓			CFU cerebellum	=
Masp-2 ab	Kasanmoen. (2)	mortality<br>clinical scores	=<br>↓					TNF blood<br>cytokines blood and brain	↓<br>=			CFU multiple organs	=
Human factor H	Kasanmoen.(3)	mortality<br>clinical scores	=<br>=					brain C5b-9<br>cytokines brain	↓<br>=			CFU lungs/spleen<br>CFU brain/blood	↓<br>=
**Antibiotic**													
DAP	Grandgirard	mortality<br>weight	=<br>↓	ABR	↓			IL-6, IL-10, MIP-1, MCP-1 CSF	↓	neuronal apoptosis<br>other histopathology	↓<br>=		
	Muri (1)	mortality<br>clinical scores	=<br>↓							cortical necrosis	↓		
	Klein (2)*	mortality<br>clinical score	=<br>=	ABR	↓<br>=			CSF leukocytes	=			CFU cerebellum	=
DAP + DEX	Klein (2)*	mortality<br>clinical score	=<br>=	ABR	↓<br>=			CSF leukocytes	=			CFU cerebellum	=
DAP + cipemastat	Muri (1)	mortality<br>clinical score	=<br>↓	ABR	↓	learning + memory<br>Morris water maze	↓	CSF cytokines	↓	hippocampal apoptosis<br>cortical necrosis	↓<br>↓	CFU CSF	↓
Doxycyclin + DAP	Muri (2)	mortality<br>clinical scores<br>weight loss	↓<br>=<br>↓	ABR	↓			CSF cytokines<br>(IL-1beta, IL-10)	↓	spiral ganglion loss<br>fibrous occlusion perilymphatic space<br>cortical necrosis	=<br>↓<br>↓	CFU CSF	↓
Doxycyclin	Meli	mortality	↓	ABR	↓					spiral ganglion loss<br>cortical damage<br>hippocampal apoptosis<br>BBB disruption	↓<br>↓<br>=<br>↓	CFU CSF	=
Rifampicin	Spreer (2)	mortality	=					CSF lactate and prostaglandin	↓	hippocampal apoptosis	↓	CSF CFU, pneumolysin, and bacterial DNA	↓
	Grandgirard	mortality<br>weight loss	=<br>=	ABR	=			CSF IL-6<br>other cytokines	↓<br>=	cortical damage<br>hippocampal apoptosis	↓<br>=		
DAP + IL-1 ab	Klein (2)	mortality<br>clinical score	=<br>=	ABR	=			CSF leukocytes	=			CFU cerebellum	=

DAP + roscovitin	Klein (2)	mortality<br>clinical score	=<br>=	ABR	=			CSF leukocytes	=			CFU cerebellum	=
**Antioxidant**													
N-acetylcysteine	Klein (1)			ABR	↓					cochlear histopathology	↓		
	Hogen	mortality<br>clinical score	=<br>=	ABR	↓	explorative activity<br>memory (T-maze)	=<br>=			cochlear histopathology	=		
MnTBAP	Kastenbauer (1)									blood-labyrinth disruption	↓		
	Kastenbauer (2)							CSF leukocytes<br>brain cytokines	↓<br>↓			CFU CSF	=
	Klein (1)					ABR	↓			cochlear histopathology	↓		
Uric acid	Kastenbauer (1)									blood-labyrinth disruption	↓		
	Kastenbauer (2)							CSF leukocytes<br>brain cytokines	↓<br>↓			CFU CSF	=
ASC	Kastenbauer (2)							CSF leukocytes<br>brain cytokines	=<br>=			CFU CSF	=
Uric acid + ASC	Kastenbauer (2)							CSF leukocytes<br>brain cytokines	↓<br>↓			CFU CSF	=
SOD	Ge (2004)			ABR	=					cochlear fibrosis	↓		
P0801	Barichello (2)							oxidative stress	↓				
**HMGB1**													
Box A protein	Hohne	clinical score	↓					CSF leukocytes<br>brain cytokines	↓<br>=	cerebral bleeding	↓	CFU	=
Ethyl pyruvate	Hohne	clinical score	↓					CSF leukocytes<br>brain cytokines	↓<br>=	cerebral bleeding	↓		
HMGB1 ab	Masouris	mortality<br>clinical score	=<br>↓					CSF leukocytes	↓	cerebral bleeding	↑	CFU brain, blood	=
HMGB1 + DEX	Masouris*	mortality<br>clinical score	=<br>=					CSF leukocytes	↓	cerebral bleeding	=	CFU brain, blood	=
HMGB1 + PAQ	Masouris	mortality<br>clinical score	=<br>=					CSF leukocytes	↓	cerebral bleeding	=	CFU brain, blood	↑
**Neurotrophin**													
BDNF	Li			ABR	↓								
	Song			ABR	=					neuronal death cortex	↓		
Neutrotrophin-3	Demel	mortality<br>clinical score	=<br>=	ABR	↓					cochlear histopathology<br>brain histopathology	↓<br>=		
LM11A-32	Zhang	mortality	=					brain leukocyte infiltration<br>cytokine expression	↓<br>↓	neurogenesis	↓		

**Matrix metalloproteinase inhibition**													
Cipemastat	Muri (1)	mortality<br>clinical score	=<br>↓							hippocampal apoptosis	↓		
B-1101	Leib (1)					learning (water maze)	↓			cortical injury<br>hippocampal apoptosis	↓<br>↓		
GM6001	Liu	clinical score	↓			learning and memory (water maze)	↓			histopathology brain	↓		
**Paquinimod**													
PAQ	Wache	clinical score	↓					CSF leukocytes and CXCL2	↓			CFU blood	=
	Masouris	mortality<br>clinical score	=<br>↓					CSF leukocytes	↓	cerebral bleeding	=	CFU blood and brain	=
**Dexamethasone**													
DEX	Addison			ABR	↓								
	Barichello (1)					memory	=	oxidative stress	=				
	Bally	mortality<br>weight loss	↑<br>↑							hippocampal regeneration	↑		
	Bhatt	ABR	=										
	Blaser (2)	mortality<br>clinical score	=<br>=					inflammatory cytokines	↓	number of apoptotic neurons	=		
	Cabellos											CFU CSF	=
	Coimbra			ABR	=					cochlear histopathology	=		
	Demel	mortality<br>motor deficits	=<br>=	ABR	=					cochlear histopathology<br>cortical injury	=<br>=		
	Demirbas			ABR	↓								
	Kasanmoen. (1)	mortality<br>clinical score	↓<br>↓										
	Kim									cochlear histopathology	↓		
	Leib	mortality<br>weight	=<br>↓			learning (water maze)	↑			hippocampal apoptosis	↑	CFU CSF	=
	Lutsar							CSF leukocytes, lactate, TNF	=			CFU CSF	=
	Rappaport			ABR	↓								
	Woehrl (2)	mortality	=							cortical injury	=		
	Zysk (1)							CSF leuckotyes and protein<br>CSF lactate	=<br>↓	hippocampal apoptosis<br>CSF neuron-specfic enolase	↑<br>↑	CSF CFU	=

**Cannaboid**													
HU-211	Bass	mortality<br>clinical score	=<br>=							cortical injury<br>brain edema<br>BBB integrity	=<br>↓<br>↓		
JWH-133	Pan	mortality<br>clinical score<br>weight	=<br>=<br>=					microglial activation	↓	coritical injury<br>hippocampal apoptosis	=<br>=		
**Other**													
anti-pneumococcal antiserum	Brandt (2)	mortality<br>clinical score	=<br>=										
betamethasone (intratympanic)	Worsoe (1)			ABR<br>otoacoustic emssions	=<br>↑					spiral ganglion density<br>tympanic fibrosis	↓<br>↑		
betamethasone (systemic)	Worsie (2)			ABR<br>otoacoustic emssions	=<br>↓					spiral ganglion density	↓		
CD18 antibody	Zysk (1)							CSF leukocytes and lactate<br>CSF protein	↓<br><br>=	hippocampal apoptosis<br>CSF neuron-specfic enolase	=<br>↓	CSF CFU	=
CXCL16 antibody	Woehrl (1)	clinical score	=					CSF leukocytes	=			CFU blood and cerebellum	=
Erythropoietin	Ertunc							CSF and serum TNF, CRP, IL-1beta	=				
Folic acid	Barichello (3)					memory	↓			oxidative stress	↓		
Fucoidin	Granert (1)							CSF leukocytes<br>CSF protein and lactate	↓<br>=				
	Granert (2)							CSF leukocytes<br>CSF IL-1 and TNF	↓<br>=				
G-CSF	Brandt (1)	mortality	=							histopathology brain	↓		
Glycerol	Blaser (1)	mortality<br>clinical score	=<br>=	ABR	=			CSF leukocytes<br>CSF MMP-9	=<br>↓	histopathology brain	=		
HspB5	Erni			ABR	=			CSF cytokines	=	cochlear histopathology	=		
Ketorolac	Rappaport			ABR	↓								
Maraviroc	Le	mortality<br>weight loss	=<br>=	ABR	=	learning and memory	=	CSF cytokines<br>microglial morphology	=<br>=	cochlear histopathology<br>CSF neurofilament light	=<br><br>=		
Melatonin	Spreer (1)	mortality	=					CSF leukocytes, protein and lactate<br>CSF prostaglandin	=<br><br>↓	hippocampal apoptosis	=	CSF CFU	=
Metformin	Muri (3)	mortality<br>clinical score	=<br>=	ABR	↓			CSF IL-1beta, IL-6 and TNF	↓	cochlear histopathology<br>cortical necrosis	↓		

								CSF IFNgamma and IL-10	=	hippocampal apoptosis	↓<br>=		
Pentoxifylline	Zysk (2)							CSF leukocytes<br>CSF lactate, protein, IL-1beta and TNF	↓<br>=				
Phosphoryl choline antibody	Gerber							CSF prostaglandin	↑	hippocampal apoptosis	↓		
Roscovitine	Kirschnek	mortality<br>clinical score	=<br>↓					CSF leukocytes	↓	cerebral hemorrhages	↓	CFU cerebellum	=
TLR2 + TLR4 antibody	Woehrl (2)	mortality	=							histopathology brain	=		
z-VAD-fmk	Braun									neuronal loss	↓		

Experimental adjunctive treatments discussed in the results are shown. A downward arrow (↓) indicates that the outcome parameter (e.g. disease severity, hearing loss, inflammation) was significantly decreased in the treated group. Equal sign (=) indicates that there was no significant difference in outcome parameter between treatment and control group. An upward arrow (↑) means that the outcome parameter was significantly higher in the treatment group. ASC = ascorbate, Ab = antibody, ABR = auditory brainstem response, BDNF = brain-derived neurotrophic factor, CFU = colony-forming units, CSF = cerebrospinal fluid, CXCL16 = chemokine ligand 16, DAP = daptomycin, DEX = dexamethasone, G-CSF = granulocyte colony-stimulating factor, HMGB1 = high mobility group box 1, HspB5 = alpha-B-crystallin, PAQ = paquinimod, SOD = superoxide dismutase, TNF = tumor necrosis factor, TLR = toll-like receptor. *compared to ceftriaxon + dexamethasone therapy
